# From Clogging Mitigation to Clogging Acceleration: Particle Deposition Under Oscillatory Flow in Microfluidic Porous Media

**DOI:** 10.1007/s11242-026-02318-0

**Published:** 2026-05-19

**Authors:** Walid Okaybi, Sophie Roman, Cyprien Soulaine

**Affiliations:** Institut des Sciences de la Terre d’Orléans, ISTO, UMR 7327, CNRS, OSUC, BRGM, Univ Orléans, 45071 Orléans, France

**Keywords:** Particle clogging, Oscillatory flow, Clogging mitigation, Porous media, Microfluidics

## Abstract

Particle deposition and clogging in porous media control the performance of many natural and engineered applications, including groundwater filtration, geothermal injection, and subsurface fluid management. Once deposits form, permeability progressively declines and flow pathways become blocked, making mitigation strategies essential for maintaining long-term operation. Oscillatory injection has been proposed as a potential strategy to delay clogging, yet how its effectiveness depends on physicochemical conditions and complex pore geometries remains poorly understood. Here, we use microfluidic experiments with colloidal suspensions to investigate how oscillatory flow modulates particle transport and clogging in tortuous porous domains. Controlled oscillatory forcing is applied over a range of frequencies while monitoring permeability evolution and deposition dynamics. Under saline conditions, oscillatory flow delays clogging and increases the injected volume before hydraulic failure with increasing frequency, consistent with electrostatic screening that promotes particle aggregation and allows cyclic pressure fluctuations to destabilize growing deposits intermittently. However, the frequency response changes with physicochemical conditions and can even reverse. As ionic strength decreases and electrostatic interactions become stronger, aggregation is suppressed, and clogging becomes increasingly governed by particle crowding and pore bridging, where higher-frequency oscillations can instead accelerate blockage. These findings demonstrate that oscillatory injection does not have a universal effect on clogging, but instead depends on the balance between hydrodynamic forcing and particle interactions. This regime-dependent behavior provides a basis for designing more effective injection strategies in porous media.

## Introduction

The transport and retention of fine particles in confined environments is a widespread phenomenon spanning multiple scales and porous media contexts. Across applications–from biological circulation (Vahidkhah et al. 2016; Patnaik et al. 1994) to groundwater transport (Ryan and Elimelech 1996; Bradford et al. 2002; Jeong et al. 2018), aquifer storage and recovery (Jeong et al. 2018), geothermal injection (Song et al. 2020), oil production (Khilar and Fogler 1983; Mungan 1965), irrigation networks (Ait-Mouheb et al. 2019), and membrane filtration and subsurface bioremediation (Keir et al. 2009; Eddaoui and Soulaine 2026)—particle accumulation progressively reduces permeability and flow capacity. Under continuous injection, deposits typically persist once formed, so restoring circulation and transport after clogging is rarely straightforward and often requires medium replacement or disruptive cleaning. This motivates the development of mitigation strategies that delay clogging and extend the lifetime of porous media, which in turn requires understanding the pore-scale mechanisms governing particle deposition and blockage.

At the pore scale, particle retention results from the competition between hydrodynamic forces (e.g., drag) and interfacial interactions (Torkzaban et al. 2007; Okaybi et al. 2025). The latter are commonly described by Derjaguin–Landau–Verwey–Overbeek (DLVO) theory (Derjaguin 1941; Verwey 1947), which represents the sum of van der Waals attraction and electrostatic double-layer forces. Varying ionic strength tunes electrostatic screening, thereby shifting the interaction balance toward adhesion at high salinity and toward repulsion in salt-free conditions (Ramachandran and Fogler 1998; Ryde et al. 1991; Torkzaban et al. 2007). Geometric confinement, quantified by the constriction-to-particle size ratio $(W/d_p)$, primarily governs which clogging mechanisms are geometrically admissible, whereas interfacial interactions control particle attachment and the stability of deposited or bridged structures. When $W/d_p<1$, sieving (straining) dominates (Delouche et al. 2021; Sauret et al. 2014; Majekodunmi and Hashmi 2022). At intermediate $W/d_p$, bridging may occur when multiple particles form a stable arch at a constriction despite being individually smaller than the entrance (Bielinski et al. 2021; Vani et al. 2022; Hsu et al. 2021; Soulaine et al. 2026). This phenomenon is most likely for $W/d_p \approx 2.5\text {--}3.33$, as reported by Sharp and Adrian (2005). For $W/d_p\gg 1$, clogging is governed by adhesive deposition through progressive surface buildup to closure (Dersoir et al. 2015, 2017; Okaybi et al. 2025). In realistic porous networks, variations in $W/d_p$, hydrodynamics, and interfacial interactions allow these mechanisms to coexist and compete. The resulting outcomes are further modulated by *i*. particle volume fraction, which sets particle arrival statistics at constrictions (Wyss et al. 2006; Fogouang Maya et al. 105158; Marin et al. 2018; Vani et al. 2022), and *ii*. pore-network tortuosity, which enhances flow-path complexity and local flux heterogeneity (Bacchin et al. 2014; Kampel et al. 2009).

Together, these clogging mechanisms lead to a decline in permeability and injectivity, progressively compromising the efficiency of the flow system. To mitigate this damage, mechanical and chemical strategies are widely implemented. Upstream filtration captures particles before they reach critical constrictions but requires additional infrastructure and maintenance (Hube et al. 2020; Hoslett et al. 2018; Aljuboury et al. 2017). Chemical treatments suppress particle depositions, mineral precipitation, and biofouling, yet involve handling risks, continuous dosing, and potential ecological impacts (Trooien et al. 1998; Bertelli et al. 2018; Wang et al. 2019). In subsurface systems, chemical–mechanical interventions such as acid fracturing can restore injectivity but must be carefully controlled to avoid re-clogging and/or environmental risks (Yudin et al. 2024; Saripalli et al. n.d.; Zang et al. 2019). These approaches are largely preventive or reactive, often costly, and may interrupt normal operation.

An alternative strategy is to modify the hydrodynamic conditions that govern clog formation and stability. In many filtration settings, backwashing/backflushing is applied as a discrete regeneration step. Flow is shortly reversed and/or hydrodynamic stresses are transiently increased after performance has degraded to dismantle partially formed deposits and restore throughput (Zsirai et al. 2012; Lohaus et al. 2020). The same principle–periodically disturbing the local force balance that stabilizes particle clogging–also motivates oscillatory forcing applied from the start of filtration. In this approach, cyclic modulation of pressure or flow rate imposes repeated hydrodynamic perturbations whose frequency and amplitude can be tuned (Dincau et al. 2020). Such forcing can perturb nascent deposits throughout operation. It has been reported to delay deposit stabilization and system-scale clogging while maintaining throughput across a range of applications, including groundwater redevelopment (Bichara 1988), irrigation systems (Zhang et al. 2017), blood microfiltration (McFaul et al. 2012; Cheng et al. 2016; Lee et al. 2018; Yoon et al. 2016), and microfluidic filtration systems (Dincau et al. 2022).

More specifically, in microfluidic colloidal filtration studies under saline conditions, deposit stability is commonly interpreted within an adhesion-dominated framework, in which attachment reflects a balance between hydrodynamic and adhesive forces (Okaybi et al. 2025; Torkzaban et al. 2007). In this regime, increasing shear can promote detachment by overcoming adhesive forces (Schwarze et al. 2019). Oscillatory forcing can intermittently exceed the detachment threshold, triggering partial-erosion events. Their effectiveness depends on the forcing amplitude and frequency, which can mitigate clogging and reveal critical operating windows with maximal mitigation, as reported in two-dimensional microfluidic channel arrays (Dincau et al. 2022). It remains unknown whether such oscillation-induced mitigation extends to heterogeneous porous domains across physicochemical regimes. This knowledge gap is especially relevant in weakly saline or salt-free conditions, where reduced ionic screening may shift clogging toward mechanically dominated bridging. In that regime, increased particle flux can accelerate near-simultaneous arrivals at constrictions and promote blockage (Agbangla et al. 2012; Sendekie and Bacchin 2016; Dressaire and Sauret 2017), yet the response of these dynamics to oscillatory forcing in heterogeneous porous networks remains largely unexplored.

To address this gap, we employ microfluidic technology to fabricate heterogeneous, tortuous porous networks that mimic subsurface geometries while enabling direct pore-scale visualization. Microfluidic devices provide high-resolution, real-time observation of transport and clogging processes in a highly reproducible manner (Manz et al. 1992; Whitesides 2006). We combine controlled variation of salinity and forcing frequency with real-time imaging and macroscopic measurements of flow-rate decline and injectivity loss. We systematically compare continuous and oscillatory injection under an imposed time-dependent driving pressure difference $\Delta P(t)$, with *t* representing time, while continuously measuring the resulting flow rate *Q*(*t*). This framework allows us to determine whether oscillatory forcing mitigates or enhances clogging in heterogeneous porous domains and to quantify the dependence of the response on physicochemical regime and frequency.

The paper is organized as follows. In Sect. 2, we describe the microfluidic porous-network geometry and fabrication, the particle suspensions, the generation and monitoring of oscillatory forcing, and the experimental and imaging protocols. In Sect. 3, we present and analyze the baseline clogging behavior under continuous injection across salt concentrations. We then analyze and compare continuous and oscillatory injection at various frequencies under saline, weakly saline, and salt-free conditions using both pore-scale visualization and macroscopic injectivity measurements. We conclude by summarizing the main findings and discussing their implications for subsurface filtration and injectivity management.

## Materials and Methods

We start by introducing the micromodel design and fabrication, together with its main hydraulic and mechanical characteristics. We then describe the particle suspensions and the method used to generate and monitor pulsatile flows. Finally, we detail the experimental procedure, including hydrodynamic characterization and the image acquisition and processing workflow used to investigate clogging under continuous and oscillatory injection, while also considering different salinity conditions.

### PDMS microfluidic devices

**Fig. 1:**
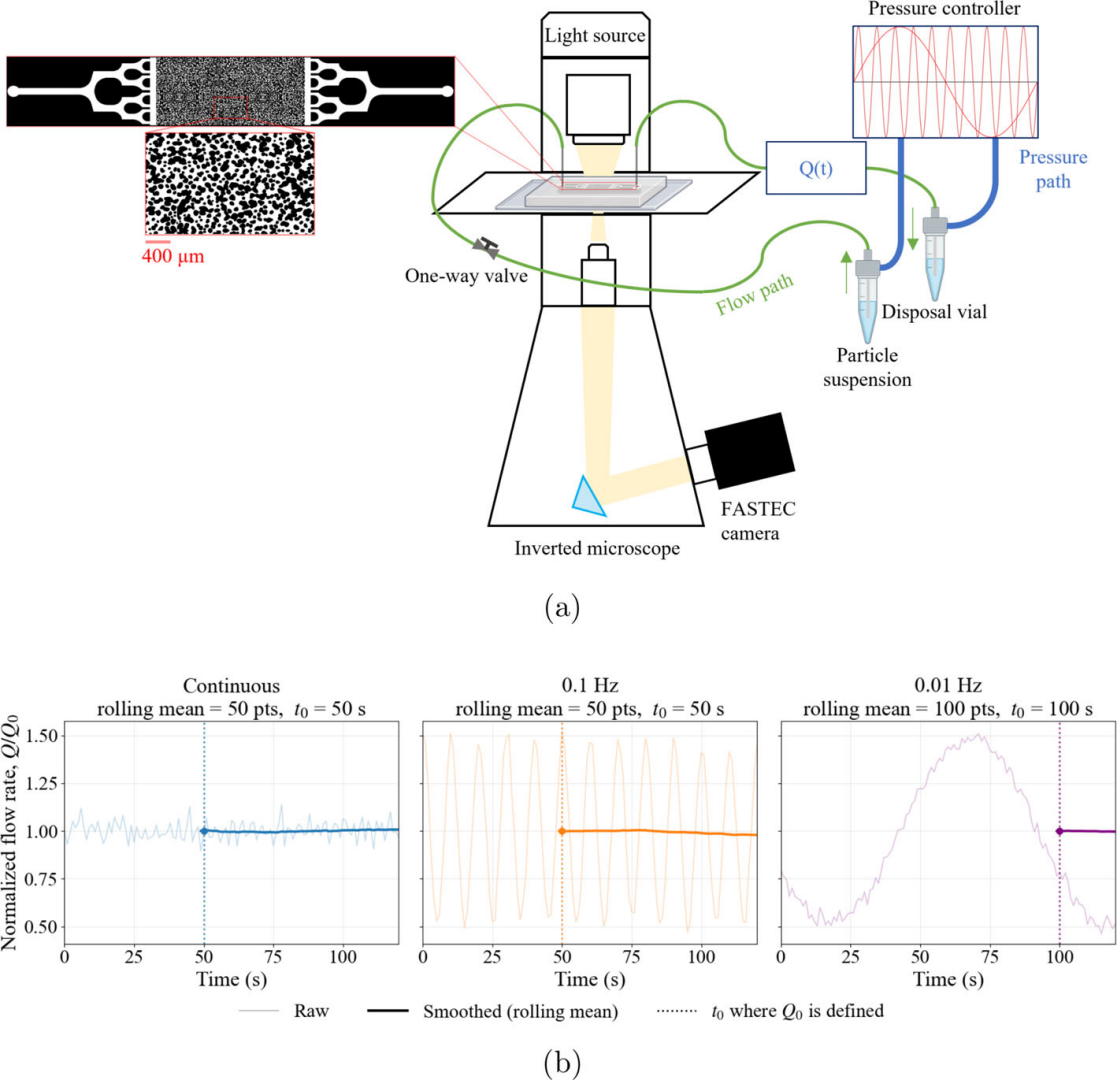
**a** Schematic of the oscillatory-flow experiments. The microfluidic device layout is shown on the left, with a magnified view of the heterogeneous porous domain highlighted below. A pressure controller applies a programmed pressure waveform at frequency *f* by pressurizing a sealed inlet reservoir, thereby driving flow through the microfluidic chip (pressure path in blue; flow path in green). The resulting volumetric flow rate *Q*(*t*) is measured downstream using a flow sensor. The chip is mounted on an inverted microscope for real-time imaging. **b** Flow-rate preprocessing and normalization procedure. Raw flow-rate signals (light curves) are smoothed using a rolling mean (thick curves). The reference flow rate $Q_0$ is computed at $t_0$ over the initial smoothing window (50 s for continuous and 0.1 Hz; 100 s for 0.01 Hz), and the signal is normalized to obtain $Q/Q_0$

We design a micromodel to reproduce the heterogeneity and tortuosity characteristic of geological porous formations, similar to the one used in our previous study on hydrodynamic pore bridging, where the layout, inlet/outlet networks, and detailed pore statistics are reported Soulaine et al. 2026. A schematic view of the device is shown in Fig. 1. The structure of the porous domain is generated using an image quilting algorithm in MATLAB, which enables the creation of large, non-repetitive porous patterns (Efros and Freeman 2023). For statistical analysis of the internal pore characteristics of the porous domain, we use MATLAB scripts to extract both the two-dimensional constriction size distribution and pore size distribution. The pore-size distribution ranges between 7 and 186 $\mu$m and follows a log-normal distribution with an arithmetic mean pore diameter of approximately $73~\mu$m, while the constriction-size distribution has an arithmetic mean throat diameter of approximately $20~\mu$m. These geometric metrics define the characteristic hydraulic length scales governing transport and clogging in the present experiments. In addition, the micromodel has porosity $\phi = 0.47$ and a permeability of $1.8 \times 10^{-12}$ m².

The geometry is replicated in poly(dimethylsiloxane) (PDMS) using standard soft-lithography techniques, resulting in a uniform channel depth of $h = 20~\mu$m. The pore volume of the porous domain is $V_p \sim 0.76~\mu \textrm{L}$. Fabrication protocols and surface-treatment procedures are also described in detail in Soulaine et al. (2026). Prior to each experiment, devices are baked at $120\,^{\circ }\textrm{C}$ for at least 3 h to ensure consistent surface properties (Dersoir et al. 2015).

PDMS compliance can affect hydraulic resistance at elevated pressures (Gervais et al. 2006; Hardy et al. 2009). Based on the device calibration reported in Soulaine et al. (2026), all experiments are conducted at $\Delta P<250~\textrm{mbar}$, where no measurable deformation-induced changes in hydraulic resistance are observed for the PDMS. Consequently, resistance variations therefore solely reflect particle transport and clogging rather than elastic deformation.

### Particle suspensions

We use monodisperse polystyrene particles with a diameter ($d_p$) of $6~\mu \text {m}$, coated with carboxyl groups, supplied as a 2.6% (*w*/*v*) stock suspension (Polysciences), where $w$ refers to weight and $v$ refers to volume. The stock suspension is diluted to a particle mass fraction of 0.3% (*w*/*w*) in a density-matched carrier fluid consisting of 22% glycerol and 78% ultra-pure water by weight. This mixture matches the particle density ($\rho _p = 1.05~\mathrm {g/cm^3}$), thereby preventing sedimentation during experiments, and has an approximate viscosity of $\mu \approx 1.9~\mathrm {mPa \cdot s}$. For experiments involving salt, NaCl is added to the density-matched carrier fluid to achieve final concentrations of 1 mM and 100 mM, where the solution remains undersaturated, and the added NaCl completely dissolves in the carrier fluid. To minimize particle aggregation, the suspension is sonicated for at least 30 min prior to each experiment.

### Oscillatory flow generation and monitoring

The oscillatory flows used in our experiments are generated as sinusoidal pressure waveforms, representing the superposition of a steady component and a harmonic oscillation. They are expressed as $\Delta P(t) = \Delta P_0 + \delta P \sin (\omega t)$, where $\Delta P_0 = 100~\text {mbar}$ is the mean pressure, $\delta P = 50~\text {mbar}$ is the oscillation amplitude, and $\omega = 2\pi f$ is the angular frequency corresponding to the imposed frequencies $f = 0.1$ and $0.01~\text {Hz}$. In all pulsatile experiments, the oscillation amplitude is kept constant, and only the forcing frequency is varied in order to investigate the effect of frequency on clogging dynamics under identical peak pressure gradients solely. The values of $\Delta P_0$ and $\delta P$ are chosen such that PDMS deformation remains negligible and no measurable damping of the oscillatory response occurs during the injection stage.

The pressure profiles are generated using an Elveflow OB1 Mk4 microfluidic flow controller (Fig. 1), capable of regulating pressure in the range 0–200 mbar. The resulting volumetric flow rate is continuously measured at the outlet using a mini CORI-FLOW (ML120V00) Coriolis mass flow meter (Bronkhorst). The device measures mass flow, with a full-scale range between 5 and 200 g/h and a minimum measurable flow of 50 mg/h (approximately 0.8 $\mu$L/min to 3.17 mL/min for a carrier-fluid density of 1.05 g/cm^3^). This range covers all conditions investigated, with an accuracy of $\pm 0.2\%$ of the reading. Because the imposed forcing introduces oscillations and measurement noise in the flow-rate signal (acquired at $1~\textrm{s}$ intervals), raw data are smoothed using a rolling-average filter implemented in Python (Fig. 1b). The window length, which corresponds to the number of data points used to compute the rolling mean (or moving average), is selected to help smooth the signal by averaging over a defined time period, effectively reducing noise and fluctuations. For continuous injection and $f = 0.1~\textrm{Hz}$, a window length of $50~\textrm{s}$ (50 data points) is used, while for $f = 0.01~\textrm{Hz}$, a window length of $100~\textrm{s}$ (100 data points) is chosen. The window length is selected to ensure consistency across continuous and oscillatory injection conditions, allowing for comparability in data smoothing. For $f = 0.1~\textrm{Hz}$, the 50-second window captures approximately 5 cycles, while for $f = 0.01~\textrm{Hz}$, the 100-second window corresponds to 1 full cycle of oscillation. The window lengths are sufficient to capture a stable baseline for normalization. The smoothed flow rate is normalized by a reference value $Q_0$ to obtain the dimensionless ratio $Q/Q_0$, where $Q_0$ is defined as the mean flow rate over the initial smoothing window (first $50~\textrm{s}$ or $100~\textrm{s}$, depending on $f$). We quantify the variability of the initial mean flow rate $Q_0$ across all performed experiments in this study and observe a maximum deviation of $7\%$ at different frequencies, which we consider acceptable for ensuring consistent initial hydraulic conditions. For example, the values of $Q_0$ in the three experimental conditions presented in Fig. 1b correspond to 5.76, 6.02, and 5.7 $\mu$L/min for continuous, $f = 0.1~\textrm{Hz}$, and $f = 0.01~\textrm{Hz}$, respectively. As illustrated in Fig. 1b, the measured flow-rate signal oscillates at the imposed frequency, with no visible phase drift and no marked attenuation during the initial stage of injection. This indicates that, over the investigated frequency range, the pressure waveform is effectively transmitted through the system and accurately captured by the flow-rate sensor. At higher frequencies, however, the flow response can become attenuated and phase-shifted relative to the imposed pressure, as reported by Dincau et al. (2022). Accordingly, the maximum forcing frequency is limited to 0.1 Hz to ensure that the flow rate remains synchronized with the imposed pressure signal.

### Flow experiments

Each experiment begins by saturating the microfluidic device with the predefined background particle-free fluid. Once full saturation is achieved, the reservoir containing the particle suspension is connected, and particle injection is initiated according to the predefined flow protocol (continuous or oscillatory pressure waveforms). The overall experimental configuration, including the pressure controller, microfluidic chip, flow sensor, and optical imaging system, is shown in Fig. 1a. When particles reach the region just upstream of the porous domain, flow-rate recording is activated downstream of the microfluidic device, and image acquisition is started using a high-speed FASTEC camera mounted on an inverted Nikon ECLIPSE Ti2 microscope. Each experimental run is terminated once the normalized flow rate reaches a critical threshold, denoted by $\theta$, such that $Q(t)/Q_0 < \theta$, with $\theta = 0.17$. This corresponds to complete clogging, where no particles from the suspension are observed flowing at the outlet. The value of $\theta$ was determined empirically from repeated experiments. The resulting $Q(t)/Q_0$ time series is used to quantify the temporal evolution of injectivity impairment. We denote by $t_\theta$ the time at which the threshold is first reached and define the cumulative injected volume up to this state as $V_\theta = \int _{0}^{t_\theta } Q(t)\,\textrm{d}t$, computed from the measured flow-rate signal.

To characterize the flow and transport regime, we evaluate the Reynolds, particle Péclet, Stokes, and Womersley numbers. Using the measured flow rates and a characteristic length scale $L = h = 20~\mu m$ (comparable to the mean constriction size reported in (Soulaine et al. 2026)), the Reynolds number $Re = \frac{\rho U L}{\mu }$ remains small for all imposed pressure drops ($Re < 10^{-2}$), confirming operation in the creeping-flow (Stokes) regime. Particle transport is quantified using the particle Péclet number, $Pe_p = \frac{U L}{D_p}$, which compares advective transport to Brownian diffusion of the suspended particles. The fluid velocity $U$ is used in the calculation of $Pe_p$ because it represents the dominant driving force of particle transport in the flow. The particle diffusivity is estimated from the Stokes–Einstein relation, $D_p = \frac{k_B T}{3 \pi \mu d_p}$, where $k_B$ is the Boltzmann constant and $T$ is the temperature (293 Kelvin). Using this relation, we obtain $D_p \sim 4 \times 10^{-14} \ \mathrm {m^2 \, s^{-1}}$. Based on the initial velocity $U_0$, we obtain $Pe_{p,0} \sim 10^5$ to $10^6$. Even at the clogging threshold ($Q/Q_0 = \theta = 0.17$), the Péclet number remains $\mathcal {O}(10^4)$ because advection dominates in regions behind the clogs, where particles continue to flow due to residual flow and fill the voids, despite no particles being observed at the outlet. The Stokes number $St = \frac{\rho _p d_p^2 U}{18 \mu L}$ remains $< 10^{-4}$, indicating negligible particle inertia and tracer-like behavior of the suspended particles. For oscillatory forcing, the Womersley number $Wo = \sqrt{\frac{\omega h^2 \rho }{\mu }}$ remains well below unity over the explored frequency range ($Wo \lesssim 10^{-2}$), indicating that viscous diffusion dominates over unsteady inertia and that the velocity field redevelops within each oscillation cycle.

### Image acquisition and deposition analysis

Image sequences are acquired at selected time points using an inverted microscope (FASTEC camera, $\times 2$ objective). Since the field of view of the camera does not span the full length of the porous domain, three adjacent regions are recorded sequentially along the main flow direction (x-direction). Each sequence is temporally averaged to highlight only the immobile (deposited) particles while removing those in motion, with image processing performed using ImageJ software to further enhance particle identification and analysis. The resulting averaged frames are then stitched into a single mosaic using a globally optimized image-stitching algorithm (Preibisch et al. 2009), yielding a full 2D representation of the porous region at the considered time. For qualitative and quantitative analysis of deposition profiles, the final stitched image at the clogging reference state $t_\theta$ is processed using a custom Python routine. The same grayscale mapping (brightness and contrast settings) is applied to all images to ensure consistent intensity values across experimental conditions. In the processed images, deposited particles correspond to low grayscale intensities, whereas the pore space and solid grains correspond to higher intensities. Accordingly, the grayscale histogram computed over the porous domain exhibits a bimodal distribution, with a narrow low-intensity peak associated with deposited particles and a broader high-intensity peak associated with the background (pore space and grains). The particle phase is isolated using a global intensity threshold, $I_{\textrm{th}}$, defined as the first local minimum of the smoothed histogram following the low-intensity peak. This criterion allows deposited particles to be identified while minimizing the inclusion of background pixels. To quantify the streamwise distribution of deposits, the reconstructed domain is divided into ten equal segments along the flow direction, and the number of particle pixels in each segment is normalized by the total number of pixels in the stitched image. This procedure yields the normalized streamwise deposition profile used to compare deposition patterns under different salinity and forcing conditions.

## Results and Discussion

In this section, we first establish the baseline clogging behavior under continuous injection for different ionic strengths. We then compare continuous and pulsatile injection to determine how oscillatory forcing modifies clogging dynamics across frequencies and salinities. Finally, we analyze pore-scale retention patterns and connect them to the observed macroscopic injectivity response. Only one representative microscopic image is shown for each condition, selected from replicate experiments performed under consistent conditions, as showing both replicates would reduce figure readability without adding qualitative information.

### Baseline clogging under continuous injection

**Fig. 2:**
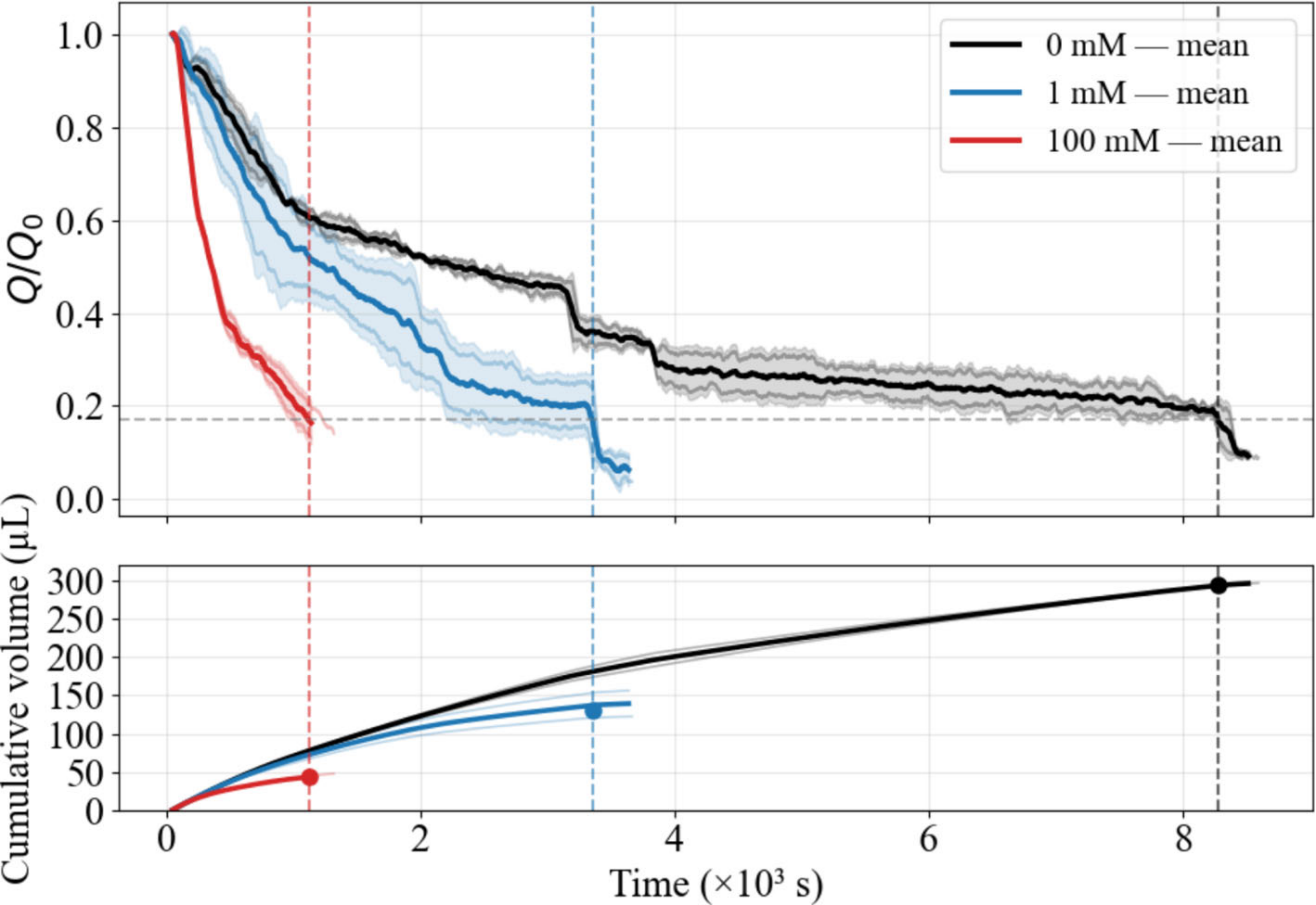
Injectivity decline under continuous forcing at different ionic strengths. Top: Normalized flow rate $Q/Q_0$ versus time for 0 mM (black), 1 mM (blue), and 100 mM (red). For each salinity, two independent replicates are shown as faint lines together with their mean (thick line). The horizontal dashed line marks the clogging threshold $Q/Q_0=\theta =0.17$, and vertical dashed lines indicate the corresponding threshold times $t_\theta$. Bottom: Cumulative injected volume $V(t)=\int _0^{t} Q(t)\,\textrm{d}t$; markers indicate $V_\theta =V(t_\theta )$. Increasing ionic strength accelerates injectivity decline and reduces $V_\theta$

We first establish a hydraulic reference by performing continuous-flow experiments at a fixed imposed pressure difference $\Delta P=100$ mbar for three ionic strengths (100 mM, 1 mM, and 0 mM). Each condition is repeated twice to assess reproducibility. Injectivity loss is monitored through the normalized flow rate $Q/Q_0$ (top panel of Fig. 2) and the cumulative injected volume (bottom panel of Fig. 2). For quantitative comparison across salinities, all conditions are evaluated at the common clogging state $Q/Q_0=\theta =0.17$, from which we extract the threshold time $t_\theta$ and the cumulative injected volume $V_\theta$.

A clear monotonic dependence on ionic strength is observed, with good reproducibility between replicas. At 100 mM (red curves), $Q/Q_0$ decreases most rapidly, yielding $t_\theta \sim 1120~\textrm{s}$ and $V_\theta =43.4\pm 4.2~\mu \textrm{L}$. Reducing ionic strength to 1 mM (blue curves) delays clogging ($t_\theta \sim 3320~\textrm{s}$) and increases the injected volume to $V_\theta =131.3\pm 32.3~\mu \textrm{L}$, corresponding to an increase of about $205\%$ relative to 100 mM. Under salt-free conditions (0 mM, black curves), the slowest injectivity decline is observed, with $t_\theta \sim 8300~\textrm{s}$ and $V_\theta =294.8\pm 0.9~\mu \textrm{L}$, i.e. about $125\%$ more than at 1 mM and about $586\%$ more than at 100 mM. This ordering is consistent with established colloidal filtration behavior: increasing ionic strength enhances electrostatic screening, promotes aggregation, and accelerates pore-spanning bridge formation, thereby reducing injectivity (Bradford et al. 2007; Torkzaban et al. 2008; Dersoir et al. 2015; Sendekie and Bacchin 2016; Dressaire and Sauret 2017; Xinqiang et al. 2019; Mesticou et al. 2016; Bennacer et al. 2017).

**Fig. 3:**
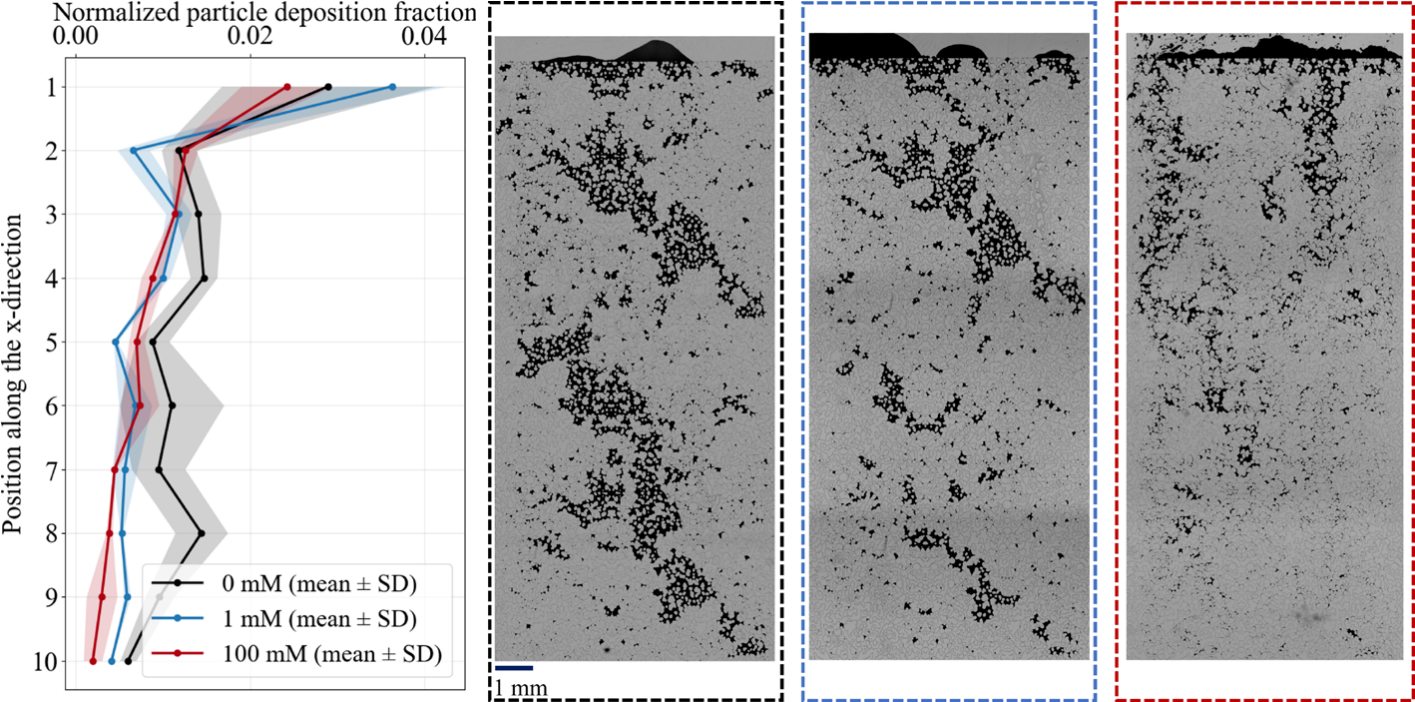
Streamwise deposition profiles at $t_\theta$ for different ionic strengths. Deposited particles are shown in black (pore space and grains in light grey). One representative stitched image is displayed for each salinity, whereas deposition profiles are obtained from two independent replicates and plotted as replicate-mean-averaged curves. Deposition is inlet-biased for all salinities, with downstream extent increasing as ionic strength decreases

To relate this injectivity trend to pore-scale deposition, we extract streamwise deposition profiles at $t_\theta$ (Fig. 3). For clarity, Fig. 3 shows one representative stitched image for each salinity, while the corresponding streamwise profiles are computed for both replicates and reported as replicate-averaged curves. The profiles show inlet-biased retention for all salinities, with deposition decreasing downstream along the porous domain. The downstream extent of deposition nonetheless increases as ionic strength decreases from 100 mM to 0 mM. At first glance, this salinity-dependent footprint may appear counterintuitive, since column-scale studies often report enhanced retention at higher ionic strength due to reduced electrostatic repulsion and stronger attachment (Bradford et al. 2007; Torkzaban et al. 2008). The key distinction here is that deposition is compared at a fixed clogging state ($Q/Q_0=\theta$), rather than after injecting the same volume. Because clogging occurs much earlier at high salinity (smaller $V_\theta$), fewer particles have the opportunity to penetrate downstream before hydraulic failure is reached.

The confinement ratio of the present porous domain further clarifies this behavior. With a mean constriction-to-particle size ratio $W/d_p\sim 3$–4, clogging occurs in a strongly confined pore-scale regime where aggregation and hydrodynamic pore bridging can coexist and directly obstruct constrictions. Under the present confined conditions, electrostatic screening at high salinity accelerates aggregation and promotes rapid upstream bridge formation (Sendekie and Bacchin 2016; Dressaire and Sauret 2017), leading to early hydraulic failure. Conversely, at low ionic strength, delayed aggregation postpones bridge formation, requiring substantially larger injected volumes to reach $Q/Q_0=\theta$. This extended injection permits deeper particle penetration and more progressive pore filling upstream of the ultimately clogged constrictions before the system is completely blocked. The larger downstream deposition extent observed at 0 mM therefore primarily reflects delayed hydraulic failure (larger $V_\theta$) rather than stronger adhesive attachment.

Having established reproducible baseline behavior under continuous injection for each salinity, we use these datasets as reference states for the subsequent oscillatory experiments. For each salinity, we then impose sinusoidal pressure oscillations at prescribed frequencies *f* (with fixed peak pressure amplitude) and assess whether pulsation mitigates clogging (increases $t_\theta$ and $V_\theta$ relative to the baseline) or instead accelerates injectivity decline.

### Mitigation under oscillatory forcing in screened conditions

We investigate the effect of oscillatory forcing in the presence of electrostatic screening (100 mM and 1 mM) by imposing sinusoidal pressure oscillations around the same mean pressure ($\Delta P_0 = 100$ mbar) with amplitude $\delta P = 50$ mbar at $f = 0.01$ and $0.1$ Hz, following the protocol described in Sect. 2.3. For each salinity, pulsatile cases are compared with the continuous-injection reference using $t_\theta$, $V_\theta$, and the mitigation factor $\Gamma (f)=V_\theta (f)/V_{\theta ,\textrm{cont}}$ (Table 1), where $V_{\theta ,\textrm{cont}}$ is the injected volume at the clogging threshold under continuous injection. Here, $\Gamma >1$ indicates mitigation and $\Gamma <1$ indicates accelerated clogging relative to continuous forcing. Unless stated otherwise, $t_\theta$ and $V_\theta$ denote replicate-averaged metrics ($n=2$), and $\Gamma$ is computed from the corresponding replicate-averaged $V_\theta$.

**Table 1:** Clogging metrics at threshold ($Q/Q_0=\theta =0.17$) for different salinity and forcing conditions

Salinity	Forcing	$t_\theta$ (s)	$V_\theta$ ($\mu$L)	$\Gamma$
100 mM	Continuous	1123	43.40	–
	0.01 Hz	1300	51.83	1.20
	0.1 Hz	2417	78.38	1.81
1 mM	Continuous	3324	131.3	–
	0.01 Hz	5203	214.6	1.64
	0.1 Hz	3541	198.0	1.51
0 mM	Continuous	8300	294.0	–
	0.01 Hz	10,707	370.0	1.26
	0.1 Hz	5315	227.5	0.77

**Fig. 4:**
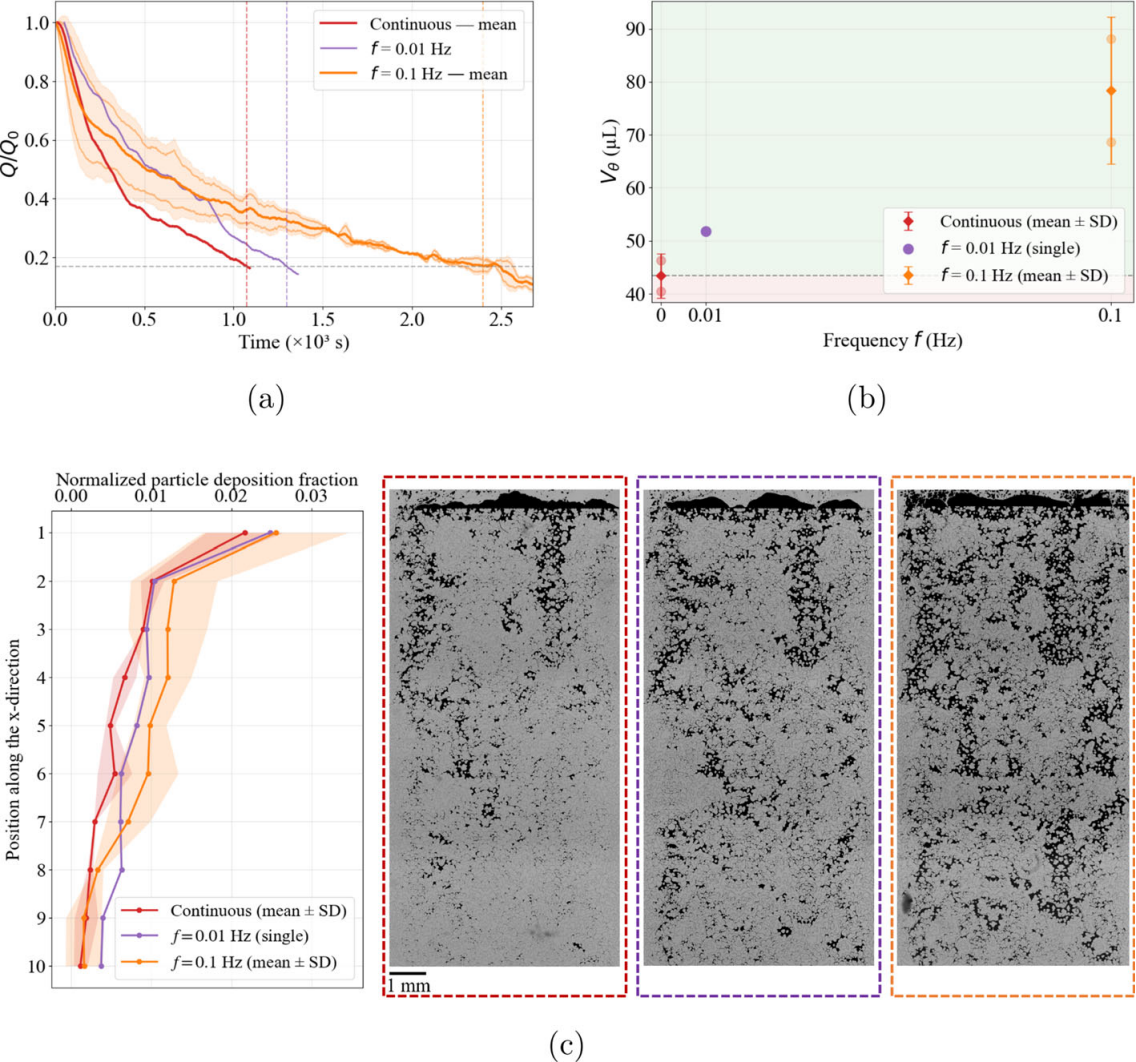
Injectivity decline (**a**) and injected volume at the clogging threshold ($V_\theta$) (**b**) for 100 mM under continuous injection (red) and oscillatory forcing at $f=0.01$ Hz (purple) and $f=0.1$ Hz (orange). The green-shaded region denotes the mitigation regime, where $V_\theta$ for both frequencies exceeds the continuous reference. (c) Streamwise deposition profiles for continuous and oscillatory forcing. For all forcing conditions, deposition is strongly inlet-biased and decreases downstream along the porous domain. Relative to continuous injection, sinusoidal forcing increases the overall downstream deposition coverage. Although local profile reversals may appear near the outlet, oscillatory forcing overall increases the downstream deposition extent compared to continuous injection

**Fig. 5:**
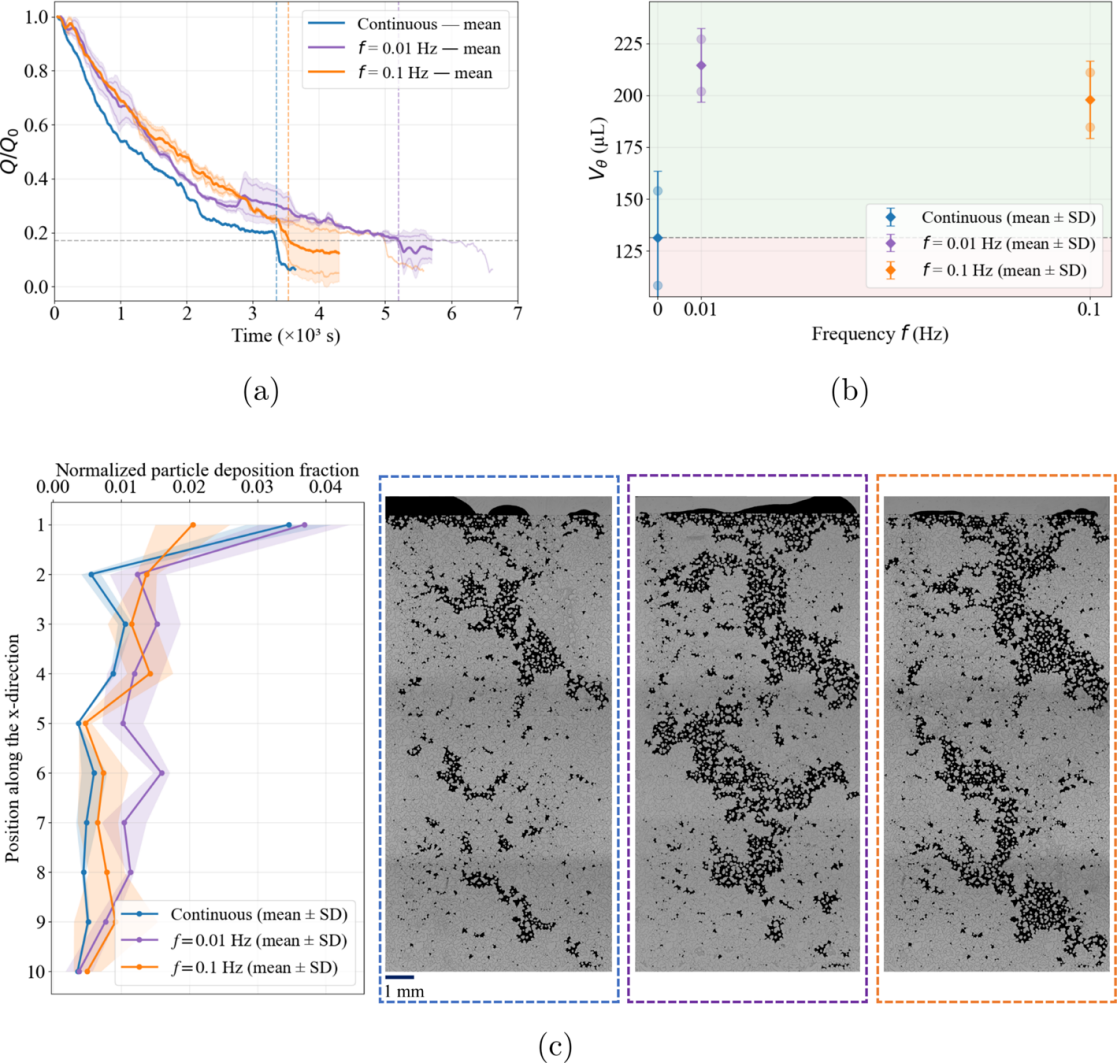
Injectivity decline (**a**) and injected volume at the clogging threshold ($V_\theta$) **b** for 1 mM under continuous injection (blue) and oscillatory forcing at $f=0.01$ Hz (purple) and $f=0.1$ Hz (orange). The $V_\theta$ for the two frequencies fall in the mitigation regime, where it exceeds the continuous reference. **c** Streamwise deposition profiles under continuous and oscillatory forcing. For all conditions, deposition remains strongly inlet-biased and decreases downstream along the porous domain. Relative to continuous injection, sinusoidal forcing increases the downstream deposition coverage with a slightly higher deposition footprint at 0.01 Hz than at 0.1 Hz

**Fig. 6:**
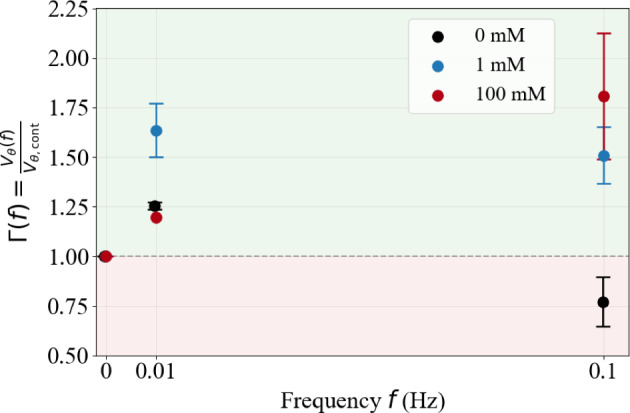
Mitigation factor $\Gamma (f)=V_{\theta }(f)/V_{\theta ,\textrm{cont}}$ versus oscillation frequency for 0 mM, 1 mM, and 100 mM. The dashed line marks $\Gamma =1$ (continuous injection); light-green shaded regions indicate mitigation ($\Gamma >1$) and light-red shaded regions indicate acceleration ($\Gamma <1$). Oscillations enhance transport at 1 mM and 100 mM for both frequencies, whereas the 0 mM case shows a regime inversion from mitigation at $0.01$ Hz to acceleration at $0.1$ Hz. The continuous-injection reference point at $\Gamma =1$ for 100 mM is slightly shifted horizontally for visualization purposes only, to avoid overlap with the corresponding points for 0 mM and 1 mM at the same reference condition. Error bars denote variability across repetitions

For both salinities and both investigated frequencies, sinusoidal forcing delays hydraulic failure relative to continuous injection (Figs. 4, 5), yielding mitigation factors $\Gamma >1$ (Fig. 6). At 100 mM (Fig. 4), the threshold time increases from $t_\theta \sim 1123$ s under continuous forcing to $\sim 1300$ s at $f=0.01$ Hz and $\sim 2417$ s at $f=0.1$ Hz. The cumulative injected volume at the clogging threshold correspondingly increases from $V_\theta = 43.40 \pm 4.15~\mu \textrm{L}$ to $\sim 52~\mu \textrm{L}$ and $78.4 \pm 13.8~\mu \textrm{L}$ at $0.01$ and $0.1$ Hz, respectively, corresponding to $\Gamma = 1.2$ and $1.81$. At $f=0.01$ Hz, replication was limited to a single experiment due to material availability and experimental time constraints; this result is therefore reported as an indicative trend rather than a statistically robust estimate. Over the tested frequencies, both $t_\theta$ and $V_\theta$ increase monotonically with *f*, indicating enhanced mitigation within this range.

At 1 mM (Fig. 5), a similar mitigation response is observed. The threshold time increases from $t_\theta \sim 3324$ s under continuous injection to $\sim 5203$ s at $f=0.01$ Hz and $\sim 3541$ s at $f=0.1$ Hz. The injected volume at threshold likewise increases from $V_\theta = 131.3 \pm 32.3~\mu \textrm{L}$ to $214.6 \pm 17.8~\mu \textrm{L}$ and $198.0 \pm 18.8~\mu \textrm{L}$, yielding $\Gamma = 1.64$ and 1.51, respectively. While both oscillatory frequencies mitigate clogging relative to continuous injection, the replicate-averaged metrics indicate only a marginal advantage of $f=0.01$ Hz over $f=0.1$ Hz.

The spatial deposition patterns at the common clogging state provide complementary insight into the mitigation mechanism. For both salinities, deposition remains predominantly inlet-biased under all forcing protocols (Figs. 4c and 5c). However, compared to continuous injection, sinusoidal forcing produces a broader downstream deposition footprint. Because deposition is evaluated at a fixed clogging state ($Q/Q_0=\theta$), the downstream footprint primarily reflects the cumulative injected volume $V_\theta$. Consequently, the larger injected volumes under oscillatory forcing translate into a redistribution of deposited particles over a larger fraction of the porous domain. Hydraulic failure is therefore delayed not by eliminating deposition, but by spatially redistributing it.

A consistent physical interpretation is that cyclic pressure modulation alters the balance between aggregation-driven accumulation and shear-induced destabilization. In screened saline conditions, DLVO energy barriers are reduced, and particle attachment is energetically favorable. During the low-pressure portion of the cycle, reduced velocities promote deposition and cluster growth in stagnation regions (Saint Vincent et al. 2016). During the subsequent high-pressure phase, transiently elevated shear stresses increase the hydrodynamic torque acting on weakly attached clusters and nascent bridges (Schwarze et al. 2019; Torkzaban et al. 2007). These stress excursions may intermittently approach the detachment threshold (Okaybi et al. 2025), limiting irreversible consolidation near pore entrances. The resulting cyclic build-up and partial erosion shift the effective hydraulic-failure condition to larger injected volumes.

Mitigation under pulsatile forcing has previously been reported in parallel-channel microfluidic arrays at a single ionic strength (100 mM) (Dincau et al. 2022). Our results in a heterogeneous, tortuous porous micromodel show the same qualitative behavior at 100 mM, including stronger mitigation at the higher of the two tested frequencies (0.1 Hz versus 0.01 Hz). Quantitatively, the sensitivity to frequency is pronounced at 100 mM: increasing *f* from 0.01 to 0.1 Hz produces a strong increase in $\Gamma$ (by $\sim 50\%$). Mitigation also persists when electrostatic screening is reduced to 1 mM, as both oscillatory cases yield $\Gamma >1$; however, the same frequency increase produces only a weak change and slightly reduces $\Gamma$ (by $\sim 8\%$). This contrast suggests that the frequency dependence of mitigation weakens, and may even reverse, as electrostatic screening is reduced.

### Frequency-driven regime inversion in salt-free conditions

**Fig. 7:**
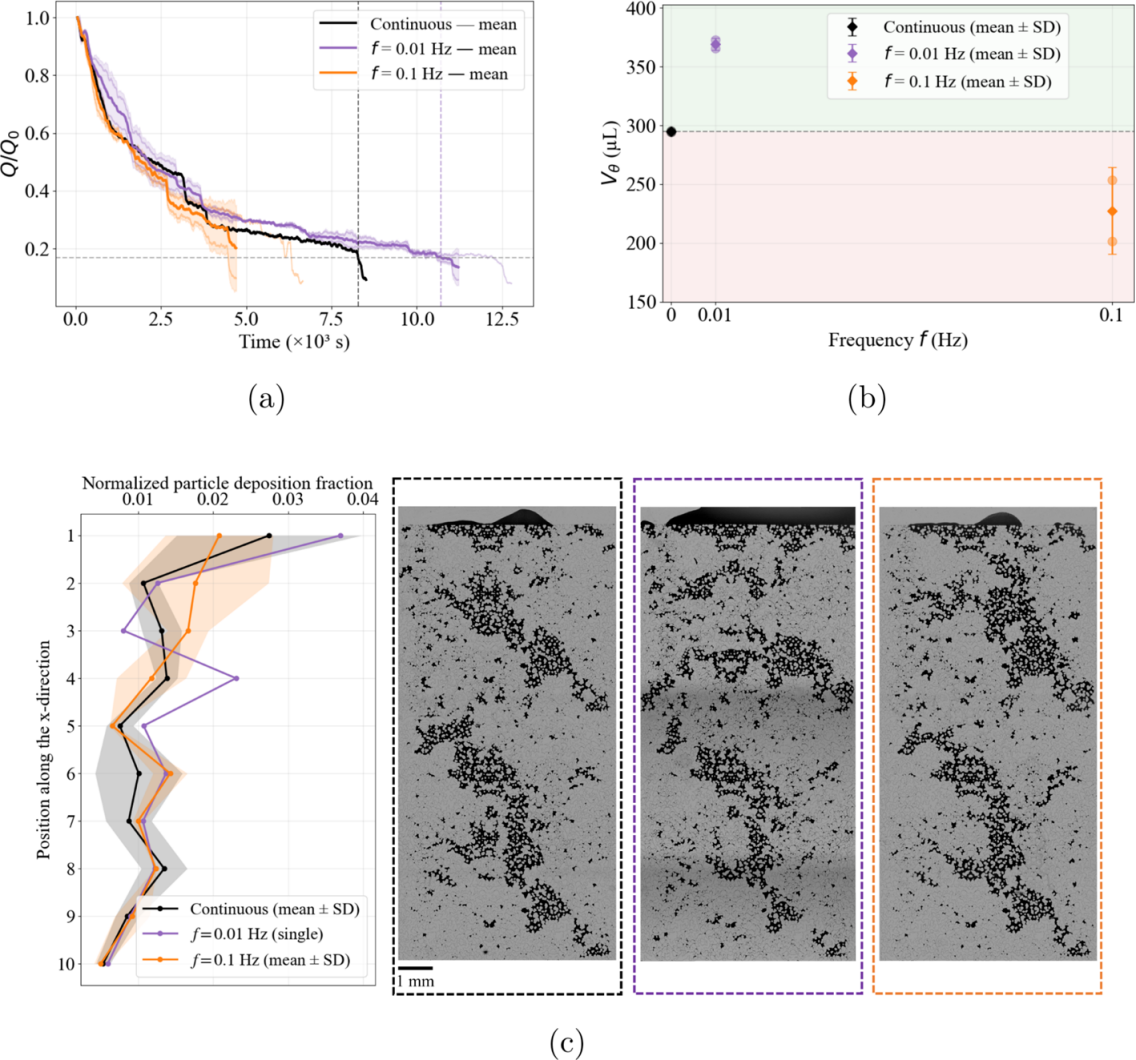
Injectivity decline (**a**) and injected volume at the clogging threshold ($V_\theta$) **b** for 0 mM under continuous injection (black) and oscillatory forcing at $f=0.01$ Hz (purple) and $f=0.1$ Hz (orange). The $f=0.01$ Hz condition falls in the mitigation regime ($V_\theta$ exceeding the continuous reference), whereas $f=0.1$ Hz falls in the clogging-acceleration regime (red-shaded region). **c** Streamwise deposition profiles under continuous and oscillatory forcing. For all conditions, deposition remains strongly inlet-biased and decreases downstream along the porous domain. No clear qualitative distinction between forcing protocols emerges, as the profiles partially overlap and intersect along several regions of the domain

In the absence of added salt, the response to oscillatory forcing differs from the finite-salinity cases (Fig. 7). Under continuous injection, the clogging state is reached at $t_\theta \sim 8300~\textrm{s}$, corresponding to an injected volume $V_\theta = 294.8 \pm 0.9~\mu \textrm{L}$. At low frequency ($f=0.01$ Hz), oscillatory forcing delays clogging relative to the continuous reference: the threshold time increases to $t_\theta \sim 10,707~\textrm{s}$, corresponding to $V_\theta = 370 \pm 5.32~\mu \textrm{L}$ and a mitigation factor $\Gamma = 1.26$. In contrast, at higher frequency ($f=0.1$ Hz) the effect reverses: the threshold time decreases to $t_\theta \sim 5315~\textrm{s}$, and the injected volume correspondingly drops to $V_\theta = 227.5 \pm 36.9~\mu \textrm{L}$, yielding $\Gamma = 0.77$. These trends are reproducible across the two independent repetitions performed at each frequency. In contrast to the finite-salinity regimes, the salt-free case shows a frequency-driven inversion, transitioning from mitigation at $f=0.01$ Hz to acceleration at $f=0.1$ Hz.

Despite the clear inversion observed in $V_\theta$, the deposition profiles at the clogging state (Fig. 7c) remain qualitatively similar and predominantly inlet-controlled, with profiles that largely overlap and occasionally intersect along the domain. This indicates that the final deposition footprint alone does not fully capture the frequency effect in the salt-free regime. Note that only one stitched image at $t_\theta$ is available for $f=0.01$ Hz due to an acquisition interruption during the second replicate. The flow-rate measurements nevertheless support the hydraulic reproducibility, and the available deposition image is representative of the observed behavior.

Sensitivity to frequency turns to be highly pronounced in the salt-free regime, where $\Gamma$ decreases by approximately 39% when the frequency increases from 0.01 Hz to 0.1 Hz, reflecting the transition from mitigation at low frequency to acceleration at higher frequency. A similar directional trend is observed at 1 mM, where increasing the frequency from 0.01 Hz to 0.1 Hz also slightly reduces $\Gamma$ (by $\sim 8\%$), although both oscillatory conditions remain within the mitigation regime. This comparison suggests that the sensitivity to frequency evolves progressively with ionic strength and reflects changes in the dominant clogging dynamics. In the salt-free limit, strong electrostatic repulsion suppresses aggregation and the system approaches a mechanically dominated regime. Given the average confinement ratio of the present micromodel ($W/d_p \sim 3$–4), clogging is then primarily governed by hydrodynamic pore bridging, where blockage arises from particle crowding and geometrically constrained collisions at pore constrictions (Dressaire and Sauret 2017; Sauret et al. 2018; Majekodunmi and Hashmi 2022).

The influence of oscillatory forcing in this mechanically dominated regime is therefore governed by the cumulative hydrodynamic loading imposed prior to blockage. Using the replicate-averaged clogging time $t_\theta$, the number of oscillation cycles experienced before reaching the clogging threshold is $N_{\textrm{cyc},\theta }=f\,t_\theta$, yielding approximately 115 cycles at 0.01 Hz and 530 cycles at 0.1 Hz. Normalizing by the replicate-averaged transported volume $V_\theta$ gives the cycling intensity

$$ \mathcal {I}_\theta =\frac{f\,t_\theta }{V_\theta }, $$

which increases from $0.30 \pm 0.03$ cycles/$\mu$L at 0.01 Hz to $2.32 \pm 0.15$ cycles/$\mu$L at 0.1 Hz. The higher frequency, therefore, imposes nearly an order-of-magnitude greater cyclic loading per unit injected volume, suggesting that the cumulative hydrodynamic loading experienced prior to clogging plays a central role in controlling the response to oscillatory forcing.

In the 0 mM case, in repulsive colloidal systems, deposition is governed primarily by particle flux density rather than adhesive stabilization. Contraction microchannel experiments under such conditions (Kim et al. 2022) show that increasing flow rate intensifies deposition at constrictions despite elevated wall shear stresses, because the amplification of particle flux under geometric confinement dominates over shear-induced removal. When particles are advected from a wider region into a narrower constriction, the reduction in cross-sectional area increases local velocity and thus particle flux density; clogging occurs once a critical crowding threshold is exceeded (Agbangla et al. 2012; Bacchin et al. 2006). In the present micromodel under repulsive conditions, the system operates close to the geometric bridging limit. Oscillatory forcing at $f=0.1$ Hz repeatedly generates transient high-velocity peaks, substantially increasing instantaneous particle flux at constrictions relative to both continuous injection and the lower-frequency case. These frequent flux amplifications increase the probability of reaching the critical crowding condition, thereby accelerating mechanically driven pore bridging. By contrast, at $f=0.01$ Hz, peak-velocity events occur less frequently, allowing nascent particle depositions to reorganize between loading cycles and delaying cumulative mechanical stabilization.

Taken together, these results demonstrate that the response to oscillatory forcing depends strongly on the physicochemical condition. While oscillations delay clogging under screened conditions, and mitigation increases monotonically with frequency, this frequency sensitivity progressively reverses as electrostatic repulsion strengthens, eventually leading to a non-monotonic regime where higher-frequency forcing accelerates clogging instead of delaying it.

## Conclusions and perspectives

Clogging in porous media affects a wide range of applications, from groundwater transport and subsurface injection to industrial filtration processes. Understanding how hydrodynamic forcing influences clog formation is therefore important for developing strategies to extend the lifetime of such processes.

In this study, we investigated how the frequency of oscillatory pressure forcing modulates clogging dynamics in heterogeneous microfluidic porous networks across different salinity conditions. Our experiments reveal that the sensitivity of clogging dynamics to oscillatory forcing evolves systematically with ionic strength. Under strongly saline conditions, oscillatory forcing delays clogging relative to continuous injection, and mitigation becomes more pronounced as the forcing frequency increases within the explored range, consistent with previous observations in parallel microfluidic channel arrays (Dincau et al. 2022). At weak salinity, oscillatory forcing relative to continuous injection still delays clogging at both tested frequencies; however, a slight inversion in performance emerges, with higher frequency marginally reducing the mitigation effect. In the salt-free limit, this trend translates into a complete inversion of the response: low-frequency forcing still delays clogging, whereas higher-frequency oscillations accelerate blockage.

These results demonstrate that oscillatory forcing is not a universally beneficial mitigation strategy. Rather, its impact depends on the dominant clogging mechanism and the underlying physicochemical regime. Under screened conditions, clogging is governed primarily by aggregation-assisted deposition, where cyclic pressure fluctuations intermittently destabilize growing deposits and delay hydraulic failure. In contrast, in the salt-free condition, strong electrostatic repulsion suppresses aggregation and shifts the system toward mechanically dominated clogging, where pore bridging is driven by particle crowding under geometric confinement. In this regime, frequent velocity peaks generated by high-frequency oscillations increase particle flux toward constrictions and accelerate blockage. This mechanistic transition explains why oscillatory forcing accelerates blockage at higher frequencies in the salt-free limit.

These findings have implications for injection processes in natural porous formations by clarifying how oscillatory forcing interacts with physicochemical conditions to control clogging dynamics. In many subsurface systems, such as geothermal reservoirs or groundwater aquifers, dissolved salts promote electrostatic screening between suspended particles. It is important to note that most natural porous formations typically deal with saline fluids, rather than saline-free fluids. Under such conditions, our results suggest that oscillatory injection may mitigate clogging and delay hydraulic failure, potentially extending the operational lifetime of injection systems. At the same time, the response to oscillatory forcing depends strongly on the physicochemical environment. While our experiments provide direct observations in heterogeneous two-dimensional porous networks, an important next step is to examine whether these regime-dependent responses persist in three-dimensional porous media. Complementary studies in simplified pore geometries would further help isolate the mechanisms controlling the transition between mitigation and acceleration under oscillatory forcing. Exploring these directions will refine predictive models and optimize flow performance in complex porous systems.

## Data Availability

Data for this article, including the injectivity decline with respect to time along with experimental images showing deposition profiles for each condition, are available in "Dataset for From clogging mitigation to clogging enhancement: particle deposition under oscillatory flow in microfluidic porous media" at https://doi.org/10.5281/zenodo.18985475.

## References

[CR1] Agbangla, G.C., Climent, É., Bacchin, P.: Experimental investigation of pore clogging by microparticles: evidence for a critical flux density of particle yielding arches and deposits. Sep. Purif. Technol. **101**, 42–48 (2012)

[CR2] Agbangla, G.C., Climent, É., Bacchin, P.: Experimental investigation of pore clogging by microparticles: evidence for a critical flux density of particle yielding arches and deposits. Sep. Purif. Technol. **101**, 42–48 (2012). 10.1016/j.seppur.2012.09.011

[CR3] Ait-Mouheb, N., Schillings, J., Al-Muhammad, J., Bendoula, R., Tomas, S., Amielh, M., Anselmet, F.: Impact of hydrodynamics on clay particle deposition and biofilm development in a labyrinth-channel dripper. Irrig. Sci. **37**(1), 1–10 (2019)

[CR4] Aljuboury, D., Palaniandy, P., Abdul Aziz, H., Feroz, S.: Treatment of petroleum wastewater by conventional and new technologies—a review. Glob. Nest J. **19**(3), 439–452 (2017)

[CR5] Bacchin, P., Aimar, P., Field, R.W.: Critical and sustainable fluxes: theory, experiments and applications. J. Membr. Sci. **281**(1–2), 42–69 (2006)

[CR6] Bacchin, P., Derekx, Q., Veyret, D., Glucina, K., Moulin, P.: Clogging of microporous channels networks: role of connectivity and tortuosity. Microfluid. Nanofluid. **17**, 85–96 (2014)

[CR7] Bennacer, L., Ahfir, N.-D., Alem, A., Wang, H.: Coupled effects of ionic strength, particle size, and flow velocity on transport and deposition of suspended particles in saturated porous media. Transp. Porous Media **118**(2), 251–269 (2017)

[CR8] Bertelli, C., Courtois, S., Rosikiewicz, M., Piriou, P., Aeby, S., Robert, S., Loret, J.-F., Greub, G.: Reduced chlorine in drinking water distribution systems impacts bacterial biodiversity in biofilms. Front. Microbiol. **9**, 2520 (2018) 30405577 10.3389/fmicb.2018.02520 PMC6205969

[CR9] Bichara, A.: Redevelopment of clogged recharge wells. J. Irrig. Drain. Eng. **114**(2), 343–350 (1988)

[CR10] Bielinski, C., Aouane, O., Harting, J., Kaoui, B.: Squeezing multiple soft particles into a constriction: transition to clogging. Phys. Rev. E **104**(6), 065101 (2021) 35030949 10.1103/PhysRevE.104.065101

[CR11] Bradford, S.A., Yates, S.R., Bettahar, M., Simunek, J.: Physical factors affecting the transport and fate of colloids in saturated porous media. Water Resour. Res. **38**(12), 63–1 (2002)

[CR12] Bradford, S.A., Torkzaban, S., Walker, S.L.: Coupling of physical and chemical mechanisms of colloid straining in saturated porous media. Water Res. **41**(13), 3012–3024 (2007) 17475302 10.1016/j.watres.2007.03.030

[CR13] Cheng, Y., Ye, X., Ma, Z., Xie, S., Wang, W.: High-throughput and clogging-free microfluidic filtration platform for on-chip cell separation from undiluted whole blood. Biomicrofluidics **10**(1) (2016) 10.1063/1.4941985 PMC4752536 26909124

[CR14] Delouche, N., Van Doorn, J., Kodger, T., Schofield, A., Sprakel, J., Tabuteau, H.: The contribution of colloidal aggregates to the clogging dynamics at the pore scale. J. Membr. Sci. **635**, 119509 (2021)

[CR15] Derjaguin, B.V.: Acta physicochim. USSR **14**, 654 (1941)

[CR16] Dersoir, B., Saint Vincent, M.R., Abkarian, M., Tabuteau, H.: Clogging of a single pore by colloidal particles. Microfluid. Nanofluid. **19**, 953–961 (2015)

[CR17] Dersoir, B., Saint Vincent, M.R., Abkarian, M., Tabuteau, H.: Clogging of a single pore by colloidal particles. Microfluid. Nanofluid. **19**(4), 953–961 (2015). 10.1007/s10404-015-1624-y.

[CR18] Dersoir, B., Schofield, A.B., Tabuteau, H.: Clogging transition induced by self filtration in a slit pore. Soft Matter **13**(10), 2054–2066 (2017) 28210719 10.1039/c6sm02605b

[CR19] Dincau, B., Dressaire, E., Sauret, A.: Pulsatile flow in microfluidic systems. Small **16**(9), 1904032 (2020) 10.1002/smll.201904032 31657131

[CR20] Dincau, B., Tang, C., Dressaire, E., Sauret, A.: Clog mitigation in a microfluidic array via pulsatile flows. Soft Matter **18**(9), 1767–1778 (2022) 35080574 10.1039/d2sm00013j

[CR21] Dressaire, E., Sauret, A.: Clogging of microfluidic systems. Soft Matter **13**(1), 37–48 (2017) 10.1039/c6sm01879c 27801463

[CR22] Dressaire, E., Sauret, A.: Clogging of microfluidic systems. Soft Matter **13**(1), 37–48 (2017). 10.1039/C6SM01879C. (**Publisher: Royal Society of Chemistry.**) 10.1039/c6sm01879c 27801463

[CR23] Eddaoui, N., Soulaine, C.: Modeling chemotaxis-biofilm competition during napl biodegradation in porous media. J. Contam. Hydrol. **279**, 104904 (2026). 10.1016/j.jconhyd.2026.104904 41806603 10.1016/j.jconhyd.2026.104904

[CR24] Efros, A.A., Freeman, W.T.: Image quilting for texture synthesis and transfer. In: Seminal Graphics Papers: Pushing the Boundaries, vol. 2, pp. 571–576 (2023)

[CR25] Fogouang Maya, L., André, L., Soulaine, C.: Numerical investigation and stochastic modeling of particle bridging undervarious flow, pore, and particle properties. Adv. Water Resour. (2025). 10.1016/j.advwatres.2025.105158

[CR26] Gervais, T., El-Ali, J., Günther, A., Jensen, K.F.: Flow-induced deformation of shallow microfluidic channels. Lab Chip **6**(4), 500–507 (2006) 16572212 10.1039/b513524a

[CR27] Hardy, B.S., Uechi, K., Zhen, J., Kavehpour, H.P.: The deformation of flexible pdms microchannels under a pressure driven flow. Lab Chip **9**(7), 935–938 (2009) 19294304 10.1039/b813061b

[CR28] Hoslett, J., Massara, T.M., Malamis, S., Ahmad, D., Van Den Boogaert, I., Katsou, E., Ahmad, B., Ghazal, H., Simons, S., Wrobel, L., et al.: Surface water filtration using granular media and membranes: a review. Sci. Total Environ. **639**, 1268–1282 (2018) 29929294 10.1016/j.scitotenv.2018.05.247

[CR29] Hsu, C.-P., Baysal, H.E., Wirenborn, G., Mårtensson, G., Wittberg, L.P., Isa, L.: Roughness-dependent clogging of particle suspensions flowing into a constriction. Soft Matter **17**(31), 7252–7259 (2021) 34318863 10.1039/d1sm00738f

[CR30] Hube, S., Eskafi, M., Hrafnkelsdóttir, K.F., Bjarnadóttir, B., Bjarnadóttir, M.Á., Axelsdóttir, S., Wu, B.: Direct membrane filtration for wastewater treatment and resource recovery: a review. Sci. Total Environ. **710**, 136375 (2020) 31923693 10.1016/j.scitotenv.2019.136375

[CR31] Jeong, H.Y., Jun, S.-C., Cheon, J.-Y., Park, M.: A review on clogging mechanisms and managements in aquifer storage and recovery (asr) applications. Geosci. J. **22**(4), 667–679 (2018)

[CR32] Kampel, G., Goldsztein, G.H., Santamarina, J.C.: Particle transport in porous media: the role of inertial effects and path tortuosity in the velocity of the particles. Appl. Phys. Lett. **95**(19) (2009)

[CR33] Keir, G., Jegatheesan, V., Vigneswaran, S.: Deep bed filtration: modeling theory and practice. Water Wastewater Treat. Technol. **8**, 263–307 (2009)

[CR34] Khilar, K.C., Fogler, H.S.: Water sensitivity of sandstones. Soc. Petrol. Eng. J. **23**(01), 55–64 (1983)

[CR35] Kim, D.Y., Jung, S.Y., Lee, Y.J., Ahn, K.H.: Effect of colloidal interactions and hydrodynamic stress on particle deposition in a single micropore. Langmuir **38**(19), 6013–6022 (2022) 35507428 10.1021/acs.langmuir.2c00237

[CR36] Lee, Y., Kim, D.-M., Li, Z., Kim, D.-E., Kim, S.-J.: Pulsatile plasma filtration and cell-free dna amplification using a water-head-driven point-of-care testing chip. Lab Chip **18**(6), 915–922 (2018) 29445802 10.1039/c7lc01328k

[CR37] Lohaus, J., Stockmeier, F., Surray, P., Lölsberg, J., Wessling, M.: What are the microscopic events during membrane backwashing? J. Membr. Sci. **602**, 117886 (2020)

[CR38] Majekodunmi, O.T., Hashmi, S.M.: Flow dynamics through discontinuous clogs of rigid particles in tapered microchannels. Sci. Rep. **12**(1), 22587 (2022) 36585430 10.1038/s41598-022-25831-w PMC9803713

[CR39] Manz, A., Harrison, D.J., Verpoorte, E.M., Fettinger, J.C., Paulus, A., Lüdi, H., Widmer, H.M.: Planar chips technology for miniaturization and integration of separation techniques into monitoring systems: capillary electrophoresis on a chip. J. Chromatogr. A **593**(1–2), 253–258 (1992)

[CR40] Marin, A., Lhuissier, H., Rossi, M., Kähler, C.J.: Clogging in constricted suspension flows. Phys. Rev. E **97**(2), 021102 (2018) 29548190 10.1103/PhysRevE.97.021102

[CR41] McFaul, S.M., Lin, B.K., Ma, H.: Cell separation based on size and deformability using microfluidic funnel ratchets. Lab Chip **12**(13), 2369–2376 (2012) 22517056 10.1039/c2lc21045b

[CR42] Mesticou, Z., Kacem, M., Dubujet, P.: Coupling effects of flow velocity and ionic strength on the clogging of a saturated porous medium. Transp. Porous Media **112**(1), 265–282 (2016)

[CR43] Mungan, N.: Permeability reduction through changes in ph and salinity. J. Petrol. Technol. **17**(12), 1449–1453 (1965)

[CR44] Okaybi, W., Roman, S., Soulaine, C.: Progressive colloidal clogging mechanism by dendritic build-up in porous media. Soft Matter (2025) 10.1039/d5sm00285k PMC12369462 40548740

[CR45] Patnaik, J.K., Das, B.S., Mishra, S.K., Mohanty, S., Satpathy, S.K., Mohanty, D.: Vascular clogging, mononuclear cell margination, and enhanced vascular permeability in the pathogenesis of human cerebral malaria. Am. J. Trop. Med. Hyg. **51**(5), 642–647 (1994) 7985757

[CR46] Preibisch, S., Saalfeld, S., Tomancak, P.: Globally optimal stitching of tiled 3d microscopic image acquisitions. Bioinformatics **25**(11), 1463–1465 (2009) 19346324 10.1093/bioinformatics/btp184 PMC2682522

[CR47] Ramachandran, V., Fogler, H.S.: Multilayer deposition of stable colloidal particles during flow within cylindrical pores. Langmuir **14**(16), 4435–4444 (1998)

[CR48] Ryan, J.N., Elimelech, M.: Colloid mobilization and transport in groundwater. Colloids Surf. A **107**, 1–56 (1996)

[CR49] Ryde, N., Kallay, N., Matijević, E.: Particle adhesion in model systems. part 14.—Experimental evaluation of multilayer deposition. J. Chem. Soc. Faraday Trans. **87**(9), 1377–1381 (1991)

[CR50] Saint Vincent, M.R., Abkarian, M., Tabuteau, H.: Dynamics of colloid accumulation under flow over porous obstacles. Soft Matter **12**(4), 1041–1050 (2016) 26573173 10.1039/c5sm01952d

[CR51] Saripalli, K.P., Bryant, S.L., Sharma, M.M.: Role of fracture face and formation plugging in injection well fracturing and injectivity decline. In: SPE Health, Safety, Security, Environment, & Social Responsibility Conference-North America, p. 52731. SPE (1999)

[CR52] Sauret, A., Barney, E.C., Perro, A., Villermaux, E., Stone, H.A., Dressaire, E.: Clogging by sieving in microchannels: application to the detection of contaminants in colloidal suspensions. Appl. Phys. Lett. **105**(7) (2014)

[CR53] Sauret, A., Somszor, K., Villermaux, E., Dressaire, E.: Growth of clogs in parallel microchannels. Phys. Rev. Fluids **3**(10), 104301 (2018)

[CR54] Schwarze, J., Grunze, M., Karahka, M., Kreuzer, H.: Attachment and detachment of particles from a surface under shear flow. J. Phys. Chem. C **123**(13), 8153–8159 (2019)

[CR55] Sendekie, Z.B., Bacchin, P.: Colloidal jamming dynamics in microchannel bottlenecks. Langmuir **32**(6), 1478–1488 (2016) 26789199 10.1021/acs.langmuir.5b04218

[CR56] Sharp, K., Adrian, R.: On flow-blocking particle structures in microtubes. Microfluid. Nanofluid. **1**(4), 376–380 (2005)

[CR57] Song, W., Liu, X., Zheng, T., Yang, J.: A review of recharge and clogging in sandstone aquifer. Geothermics **87**, 101857 (2020). 10.1016/j.geothermics.2020.101857

[CR58] Soulaine, C., Okaybi, W., Maya, L.F., Le Trong, E., Roman, S.: Permeability impairment by hydrodynamic pore bridging: Probabilistic pore-network modeling and microfluidic experiments. Int. J. Rock Mech. Min. Sci. **200**, 106423 (2026)

[CR59] Torkzaban, S., Bradford, S.A., Walker, S.L.: Resolving the coupled effects of hydrodynamics and dlvo forces on colloid attachment in porous media. Langmuir **23**(19), 9652–9660 (2007) 17705511 10.1021/la700995e

[CR60] Torkzaban, S., Bradford, S.A., Genuchten, M.T., Walker, S.L.: Colloid transport in unsaturated porous media: The role of water content and ionic strength on particle straining. J. Contam. Hydrol. **96**(1–4), 113–127 (2008) 18068262 10.1016/j.jconhyd.2007.10.006

[CR61] Trooien, T.P., Alam, M., Freddie, R.L., Freddie, R.: Filtration and maintenance considerations for sdi systems (1998)

[CR62] Vahidkhah, K., Balogh, P., Bagchi, P.: Flow of red blood cells in stenosed microvessels. Sci. Rep. **6**(1), 28194 (2016) 27319318 10.1038/srep28194 PMC4913314

[CR63] Vani, N., Escudier, S., Sauret, A.: Influence of the solid fraction on the clogging by bridging of suspensions in constricted channels. Soft Matter **18**(36), 6987–6997 (2022). 10.1039/d2sm00962e 36069637 10.1039/d2sm00962e

[CR64] Verwey, E.J.W.: Theory of the stability of lyophobic colloids. J. Phys. Chem. **51**(3), 631–636 (1947) 10.1021/j150453a001 20238663

[CR65] Wang, Y.-H., Wu, Y.-H., Tong, X., Yu, T., Peng, L., Bai, Y., Zhao, X.-H., Huo, Z.-Y., Ikuno, N., Hu, H.-Y.: Chlorine disinfection significantly aggravated the biofouling of reverse osmosis membrane used for municipal wastewater reclamation. Water Res. **154**, 246–257 (2019) 30798179 10.1016/j.watres.2019.02.008

[CR66] Whitesides, G.M.: The origins and the future of microfluidics. Nature **442**(7101), 368–373 (2006) 16871203 10.1038/nature05058

[CR67] Wyss, H.M., Blair, D.L., Morris, J.F., Stone, H.A., Weitz, D.A.: Mechanism for clogging of microchannels. Phys. Rev. E **74**(6), 061402 (2006) 10.1103/PhysRevE.74.061402 17280068

[CR68] Xinqiang, D., Yalin, S., Xueyan, Y., Ran, L.: Colloid clogging of saturated porous media under varying ionic strength and roughness during managed aquifer recharge. J. Water Reuse Desalination **9**(3), 225–231 (2019)

[CR69] Yoon, Y., Kim, S., Lee, J., Choi, J., Kim, R.-K., Lee, S.-J., Sul, O., Lee, S.-B.: Clogging-free microfluidics for continuous size-based separation of microparticles. Sci. Rep. **6**(1), 1–8 (2016) 27198601 10.1038/srep26531 PMC4873827

[CR70] Yudin, A., Eltaher, E., AlYaseen, A., Al-Jalal, Z., Faisal, M.: Water wells injectivity enhancement via hydraulic fracturing in open hole completions. In: SPE Annual Caspian Technical Conference, pp. 011-002005. SPE (2024)

[CR71] Zang, A., Zimmermann, G., Hofmann, H., Stephansson, O., Min, K.-B., Kim, K.Y.: How to reduce fluid-injection-induced seismicity. Rock Mech. Rock Eng. **52**(2), 475–493 (2019)

[CR72] Zhang, L., Wu, P., Zhu, D., Zheng, C.: Effect of pulsating pressure on labyrinth emitter clogging. Irrig. Sci. **35**(4), 267–274 (2017)

[CR73] Zsirai, T., Buzatu, P., Aerts, P., Judd, S.: Efficacy of relaxation, backflushing, chemical cleaning and clogging removal for an immersed hollow fibre membrane bioreactor. Water Res. **46**(14), 4499–4507 (2012) 22709984 10.1016/j.watres.2012.05.004

